# Infrared Thermography to Evaluate Heat Tolerance in Different Genetic Groups of Lambs

**DOI:** 10.3390/s150717258

**Published:** 2015-07-16

**Authors:** Concepta McManus, Eliandra Bianchini, Tiago do Prado Paim, Flavia Gontijo de Lima, José Braccini Neto, Marlos Castanheira, Geisa Isilda Ferreira Esteves, Caio Cesar Cardoso, Vanessa Calderaro Dalcin

**Affiliations:** 1 Faculdade de Agronomia e Medicina Veterinária, Universidade de Brasília, Brasília 70910-900, DF, Brasil; E-Mails: concepta.mcmanus@ufrgs.br (C.M.); eliandrabianchini@gmail.com (E.B.); isildinha@gmail.com (G.I.F.E.); cajug29@gmail.com (C.C.C.); 2 Departamento de Zootecnia, Universidade Federal do Rio Grande do Sul, Porto Alegre 91540-000, Brasil; E-Mails: jose.braccini@ufrgs.br (J.B.N.); vanessacdalcin@yahoo.com.br (V.C.D.); 3 Instituto Federal de Educação, Ciência e Tecnologia Goiano–Campus Iporá, Iporá 76200-000, Brasil; 4 Escola de Veterinária, Universidade Federal de Goiás, Goiânia, Goiás Paulo 74690-900, Brasil; E-Mails: flaviamedvet@yahoo.com.br (F.G.L.); castanheira_m@hotmail.com (M.C.)

**Keywords:** adaptation, naturalized breeds, genetic resources, temperature, tropical

## Abstract

Heat stress is considered a limiting factor for sheep production. We used information from physiological characteristics linked to heat tolerance to determine whether infrared thermography temperatures were able to separate groups of animals and determine the most important variables in this differentiation. Forty-eight four-month-old male lambs from eight genetic groups were used. Physiological (rectal temperature–RT, heart rate–HR, respiratory rate–RR) and blood traits, infrared thermography temperatures, heat tolerance indices, body measurements, weight and carcass traits were measured. Statistical analyses included variance, correlations, factor, discrimination and regression. Observing the correlations between physiological characteristics (RT, RR and HR) with temperatures measured by infrared thermography, regions for further studies should include the mean temperature of flank, nose and rump. Results show that there are strong relationships between thermograph measurements and RR, RT and HR in lambs, which are suggested to be directly correlated with heat tolerance capacity of the different genetic groups evaluated in this study. The assessment of body surface temperature measured by the thermograph could be used as a noninvasive tool to assess heat tolerance of the animals.

## 1. Introduction

Heat stress is a limiting factor for sheep production in tropical or warm regions. It has been shown that it is necessary to select animals adapted to harsh environments, often outside their physiological comfort zone, for production purposes [1]. Recently, several terminal sire ram breeds have been introduced into tropical areas of Brazil for use in crossbreeding systems. The environmental origin of the breeds is frequently different from those where they are expected to produce and reproduce [2]. Therefore, crossing local breeds may produce a non-optimal adaptation to the local environmental conditions, and finally contribute to important economic losses.

Breed choice for sheep production in the tropics should take into account adaptation to the environment and the effects of climate on the physiological parameters and performance of animals [3]. In recent years, with climate changes, research on animal welfare has intensified in an attempt to minimize economic losses due to the effects of climate on animal production in the tropics [4]. Animal welfare is a demand for any breeding system that wants to be ethically defensible and socially acceptable [5].

Animal breeding must respond quickly to climate change under predicted acceleration of global warming [6]. Global warming will anticipate changes in the vegetation of tropical regions and in some places the pasture area may decrease by up to 50% [7]. The effects of climate on animal production are therefore of interest to agricultural and public policy makers [8].

Sheep are animals that are well adapted to diverse ecosystems. Temperature and relative humidity are factors that can influence the rearing of these animals. At high temperatures and high radiation, they have difficulty in losing heat and, thereby, regulation of their internal temperatures [1]. For ruminants raised in tropical conditions, in general, the thermolysis mechanism is considered more effective than evaporation since the ambient air temperature tends to be close to skin surface temperature, neutralizing heat exchange by conduction and convection [9]. Several physiological characteristics have been used to evaluate the suitability of the animal in adverse climates [2].

The aim of this study was to evaluate the use of information from physiological characteristics related to heat tolerance, blood parameters, temperatures measured with infrared thermography, morphometric measurements and carcass traits in lambs of different genetic groups. Moreover, we aimed to determine the usefulness of infrared thermography to evaluate heat tolerance and the usefulness of the traits measured to separate genetic groups and determine the most important variables in the differentiation of these groups for heat tolerance.

## 2. Experimental Section

Forty eight four-month-old male lambs from eight genetic groups were used: 50% East Friesian × 50% Santa Inês (EFSI); 50% Primera × 50% Santa Inês (PRSI); 87.5% Poll Dorset × 12.5% Santa Inês (87PDSI); 100% Santa Inês (SI); 50% Dorper × 50% Poll Dorset (DOPD); 50% Poll Dorset × 50% Santa Inês (PDSI); 50% White Dorper × 50% Poll Dorset (WDPD); 75% Poll Dorset × 25% Santa Inês (75PDSI).

The animals were fed Tifton 85 (*Cynodon* spp.) hay *ad libitum* and 300 g/day of a concentrate based on corn, soybean and minerals. During the experimental period, air temperature (AT) and relative humidity (RH), black globe temperature in the sun (BGTsun) and shade (BGTsd) and wind speed (WS) were obtained using an environmental monitoring station.

Rectal temperature (RT), respiratory rate (RR) and heart rate (HR) were measured on two occasions: at 6:30 a.m. and 12:00 p.m. with six repetitions. Between the two collections, the animals were kept in open sunlight. RT was measured using a digital clinical thermometer inserted near the rectal wall of animal, at a depth of approximately 3.5 cm. RR and HR were measured using a stethoscope.

The indices of heat tolerance calculated included:

Temperature and humidity index (THI): THI = Tdb °C−{(0.31−0.31RH)(Tdb °C−14.4)}
(1)

where: Tdb: dry bulb temperature (air temperature) and RH: relative air humidity (%);

Rauschenbach−Yerokhin [10]: ITC = 1.0 AT − 20 d + 60
(2)

where: AT = air temperature and d = difference between 6 a.m. and 12 p.m. rectal temperatures and a value closer to 100 indicates a better adapted animal;

Ibéria or Rhoad test: CTC = 100 − [18 (RT − 39.1)]
(3)

where CTC = heat tolerance coefficient; 100 = maximum efficiency in maintaining body temperature below 39.1 °C; 18 = constant; RT = mean final rectal temperature; 39.1 °C = normal mean rectal temperature for sheep [11]. Value closer to 100 indicates a better adapted animal;

Benezra test: CA = RT/39.1 + RR/27
(4)

where: CA = adaptability coefficient; RT = rectal temperature in °C; RR = respiratory rate per minute; 39.1 °C = normal mean rectal temperature for sheep; 27 = normal mean respiratory rate for sheep [11]. Value close to two means animals that are better adapted;

Adapted from Baccari Jr. [12]: ICTI = 10 − (RT2 − RT1)
(5)

where: ICTI = Index of capacity of tolerance to insolation; 10 = maximum efficiency in maintaining body temperature; RT2 = mean body temperature at 12 h; and RT1 = mean body temperature at 6.5 h. Value closer to 10 indicates better adapted animals.

Hematologic parameters in both periods (morning and afternoon) were determined from blood samples using a Mindray BC 2800 vet apparatus and the microhematocrit technique was used for determination of plasma fibrinogen. Absolute red blood cell (RBC) indices were calculated: mean corpuscular volume (MCV), red cell distribution width (RDW), mean corpuscular hemoglobin (MCH) and mean corpuscular hemoglobin concentration (MCHC).

Surface temperatures were measured with infrared thermography (FLIR i-Series^®^ system) and analyzed using QuickReport^®^ software. Temperatures were measured in the regions of the nose, head (a point next to brain location), neck, axilla, stifle and croup of animals, as well as a mean temperature forflank, following the same temperature points measured by Paim *et al.* [13].

Body measurements, such as wither height (WH); heart girth (HG); body length (BL); back length (LB) and skin thickness (ST), were measured. Animals were then slaughtered and cold carcass weight (CW) as well as commercial cuts including neck, belly, shoulder, shank, rib and back were measured according to Silva Sobrinho [14]. Percentages of cuts were calculated dividing double the cut weight by CW.

Data were analyzed using the SAS^®^ software using MIXED procedure for analysis of variance with an unstructured covariance matrix for the significant variables selected by the BIC criterion. Stepwise multivariate regression (REG) was used to test the use of infrared thermography in determining changes in physiological variables. Data was separated into physiological traits with indices and carcass traits with body size measurements, and these were analyzed separately for the morning and afternoon. A factor analysis (FACTOR) was used to examine the relationship between the variables. Clusters (FASTCLUS) were formed and a discriminant analysis (DISCRIM) was used to predict the traits that differentiated between genetic groups. Normality was tested using the Univariate procedure. Non-normal characteristics were transformed: percentages and proportions by arcsine; counts by square root and others using Tukey’s Ladder of Powers.

## 3. Results

The temperature and humidity index (THI) in the morning classified the environment as being moderately stressful (22.69). In the afternoon, the animals were subjected to extremely severe stress (ranging from 26.69 to 28.78). Based on the scale proposed by Silanikove [15], 23.73% of the sheep on this experiment were under high stress and 51.75% under very high stress in the afternoon, *i.e.*, the animals were using RR as a means of heat dissipation to maintain their homeothermy (Table 1). The maximum RR in sheep during the experiment was 192 mov·min^−1^.

**Table 1:** Scaling of respiratory rate (RR) of sheep in this experiment, according to the period of the day.

RR for Sheep *	Stress Level	Morning % Animals	Afternoon % Animals
<40 mov·min^−1^	Absence	84.72	0.72
40–60 mov·min^−1^	Low	11.81	14.36
61–80 mov·min^−1^	Medium-High	2.08	10.13
81–120 mov·min^−1^	High	0.69	23.04
121–192 mov·min^−1^	Very High	-	51.75
>193 mov·min^−1^	Severe	-	-

* Adapted from Silanikove [15].

The RT in the afternoon (39.95 °C) was higher (*p* < 0.0001) than in the morning (38.89 °C). Table 2 shows that the PRSI genetic group had the lowest RT temperature (39.43 °C) in the afternoon, differing from the other genetic groups. In the morning, the SI had the lowest RT (38.27 °C) while WDPD showed higher RR in the afternoon than EFSI, and EFSI showed higher RR than 75PDSI, PDSI, PRSI and SI in the morning. HR in the morning also showed EFSI with higher values than 75PDSI, PDSI, SI and WDPD.

**Table 2:** Means of the genetic groups physiological characteristics (HR, RR and RT)

Genetic Group	HR		RR		RT	
	M	A	M	A	M	A
75PDSI	66.66 ^dc^	72.44	27.77 ^b^	122.44 ^ab^	38.81 ^b^	39.98 ^b^
87PDSI	80.22 ^ab^	76.50	33.77 ^ab^	119.0 ^ab^	39.32 ^a^	40.08 ^ab^
DOPD	73.77 ^abc^	79.33	32.88 ^ab^	124.89 ^ab^	39.30 ^a^	40.36 ^a^
EFSI	81.55 ^a^	79.55	39.77 ^a^	93.78 ^b^	38.77 ^b^	39.80 ^b^
PDSI	62.66 ^d^	74.66	28.22 ^b^	134.22 ^ab^	38.86 ^b^	39.94 ^b^
PRSI	75.33 ^abc^	74.85	25.77 ^b^	100.50 ^ab^	38.72 ^b^	39.43 ^c^
SI	70.00 ^bcd^	78.82	27.33 ^b^	112.24 ^ab^	38.27 ^c^	39.89 ^b^
WDPD	65.77 ^dc^	76.66	33.55 ^ab^	138.22 ^a^	39.05 ^ab^	40.07 ^ab^
Mean	71.99 ^a^	76.60 ^b^	31.52 ^a^	118.16 ^b^	38.88 ^a^	39.94 ^b^

Means followed by different letters by column are significantly different using SNK test (*p* < 0.05). HR–heart rate; RR–respiratory rate; RT–rectal temperature; M–morning; A–afternoon. PD: Poll Dorset; PR: Primera; EF: East Friesian; SI:Santa Inês; WD: White Dorper; DO: Dorper.

The numbers of erythrocytes were higher in SI (13.14 × 10^6^ mm^−3^) and DOPD (12.87) than WDPD (11.24) and 75PDSI (11.22). The same pattern was seen in hemoglobin concentration. The SI had higher MCV (31.50) than PRSI (27.33), WDPD (26.76) and 75PDSI (26.75). The genetic group DOPD had the highest MCHC and EFSI genetic group showed higher RDW. The WDPD had higher fibrinogen (477.78) than EFSI (211.11).

AT, RH, WS, BGTsun and BGTsd climatic variables affected (*p* < 0.05) the thermography measurements, which may infer that environmental conditions influenced the increase of the animal's body temperature. In general, the EFSI genetic group obtained the highest thermographic measures, and the lowest were observed in the 87PDSI genetic group (Table 3). The nose measurements did not differ between genetic groups.

**Table 3:** Mean values of the temperatures obtained with the thermograph in their respective regions, according to the sheep genetic group.

Genetic Group	Rump (°C)	Head (°C)	Axilla (°C)	Neck (°C)	Groin (°C)	Nose (°C)	Flank (°C)
75PDSI	33.84 ^a^	33.61 ^abc^	34.08 ^ab^	32.71 ^b^	33.68 ^bc^	33.10	33.62 ^a^
87PDSI	31.95 ^b^	32.80 ^c^	33.10 ^bc^	31.29 ^c^	32.99 ^dc^	32.86	32.00 ^b^
DOPD	33.23 ^ab^	34.26 ^ab^	35.01 ^a^	32.95 ^b^	34.53 ^ab^	33.19	33.25 ^a^
EFSI	33.01 ^ab^	34.49 ^a^	35.12 ^a^	34.26 ^a^	35.29 ^a^	33.52	34.02 ^a^
PDSI	34.11 ^a^	34.01 ^ab^	34.94 ^a^	33.14 ^b^	35.11 ^ab^	32.78	34.04 ^a^
PRSI	32.68 ^ab^	33.80 ^ab^	34.39 ^a^	33.67 ^ab^	34.97 ^ab^	32.69	33.39 ^a^
SI	33.69 ^a^	33.63 ^abc^	35.16 ^a^	34.64 ^a^	35.85 ^a^	32.68	34.42 ^a^
WDPD	32.66 ^ab^	33.39 ^bc^	32.64 ^c^	31.44 ^c^	32.03 ^d^	32.60	32.15 ^b^

Means followed by different letters by column are significantly different using SNK test (*p* < 0.05). PD: Poll Dorset; PR: Primera; EF: East Friesian; SI: Santa Inês; WD: White Dorper; DO: Dorper.

No statistical difference was observed comparing genetic groups using the Benezra test (Table 4). The SI had the lowest index for Baccari Jr. (8.32) and Rauschenbach-Yerokhin (54.48), which indicate poorest heat tolerance.

**Table 4:** Mean values of the adaptability tests for genetic groups of sheep in Central-West Brazil.

Genetic Group	Rauschenbach-Yerokhin	Ibéria	Benezra	Baccari Jr. Adapted
75PDSI	64.58 ^a^	88.74 ^ab^	3.75	8.82 ^a^
87PDSI	73.28 ^a^	88.30 ^ab^	3.70	9.26 ^a^
DOPD	66.78 ^a^	84.30 ^b^	3.89	8.93 ^a^
EFSI	66.51 ^a^	89.17 ^ab^	3.42	8.92 ^a^
PDSI	66.33 ^a^	89.35 ^ab^	3.96	8.91 ^a^
PDWD	67.67 ^a^	88.35 ^ab^	4.15	8.98 ^a^
PRSI	72.70 ^a^	92.40 ^a^	3.22	9.23 ^a^
SI	54.48 ^b^	83.86 ^b^	3.50	8.32 ^b^

Means followed by different letters by column differ using SNK test (*p* < 0.05). PD: Poll Dorset; PR: Primera; EF: East Friesian; SI:Santa Inês; WD: White Dorper; DO: Dorper.

The predictive regression equations (Table 5) show that flank, nose, rump and neck temperatures are the best predictors of physiological responses to heat. The inclusion of air temperature or humidity in the equation increased R^2^ by approximately 1% while air temperatures or humidity alone gave predictive equations approximately 20% lower than those shown here. 

**Table 5:** Multiple regressions for rates of heat tolerance and physiological variables using infrared thermography.

Variables	Multivariate Regression Equation	R^2^
Ibéria	156.43 − 3.24 nose *** + 2.05 neck *** − 0.02 rump^2^ ***	0.50
Benezra	49.95 − 2.58 nose ** + 0.04 nose² ** + 0.02 rump² **	0.50
RY	873.47 − 1.21 flank ** − 49.40 nose ** + 0.75 nose² **	0.30
Baccari	−30.42 − 0.07 flank **+ 2.56 nose *** −0.03 nose² ***	0.33
RR	−122.14 + 0.102 nose² * + 0.071 rump²*	0.50
HR	91.46 + 0.03 nose² **	0.05
RT	35.96 + 0.18 nose *** − 0.11 neck *** + 0.001 rump² ***	0.50

RY: Rauschenbach − Yerokhin; RR: respiratory rate; HR: heart rate; RR: rectal temperature; *** *p* < 0.0001; ** *p* < 0.001; * *p* < 0.05.

The correlations between the temperatures measured by infrared thermography and RR were high and positive for the flank (r = 0.72), rump (0.75) and nose (0.68). Rectal temperature was correlated positively with thermal image temperatures for flank (r = 0.54), head (0.58), nose (0.72) and rump (0.62). RR and RT had a correlation of 0.70. The infrared temperatures had high and positive correlations with Benezra: flank (r = 0.72), head (0.66), nose (0.68), neck (0.63) and rump (0.75). The Iberia test showed correlations with nose (r = −0.72) and rump (−0.62). The flank temperature had medium correlations RY (0.33) and Baccari (−0.39) tests.

**Figure 1:**
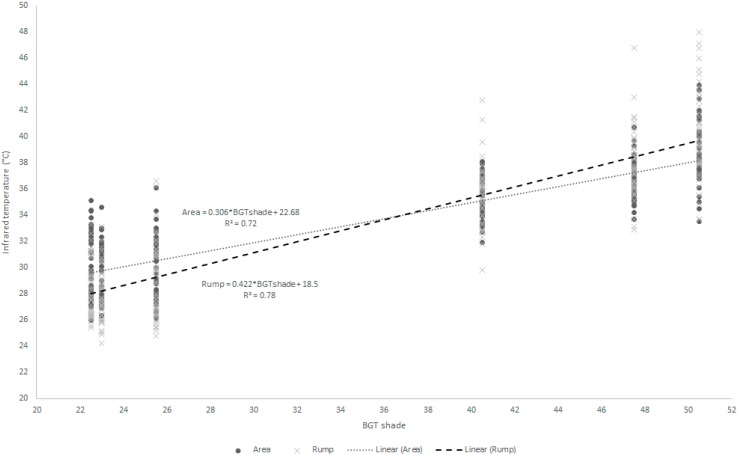
Data distribution and regression equation between infrared temperatures points (rump and flank) and black globe temperature under shade (BGTsd).

The infrared temperatures had significant correlations with environmental factors. The AT had high and positive correlations with all surface temperatures (0.54 < r < 0.82). The BGT under sun and shade had high and positive correlations with all surface temperatures (0.58 < r < 0.88). In both, the axilla had the lowest correlation and the rump and the flank had the highest. The regression equations (Table 6 and Figure 1) show the relation between environmental factors, such as air temperature (AT), air relative humidity (RH), wind speed (WS) and BGT under sun (BGTsun) and shade (BGTsd), and the infrared temperatures. The regressions showed the BGTsd explaining a high proportion of variation in infrared temperatures, mainly the flank and rump points (Figure 1).

**Table 6:** Multiple regressions for infrared thermography data using environmental factors.

Variables	Multivariate Regression Equation	R^2^
Neck	24.96 + 0.23 BGTsd ***	0.59
Groin	27.81 + 0.187 BGTsd ***	0.39
Axilla	29.31 + 0.139 BGTsd ***	0.34
Head	58.60 + 1.14 WS * + 0.172 BGTsd *** − 2.249 AT * + 0.04 AT² *	0.33
Nose	27.79 + 1.40 WS ** + 0.144 BGTsd ***	0.59

**Figure 2:**
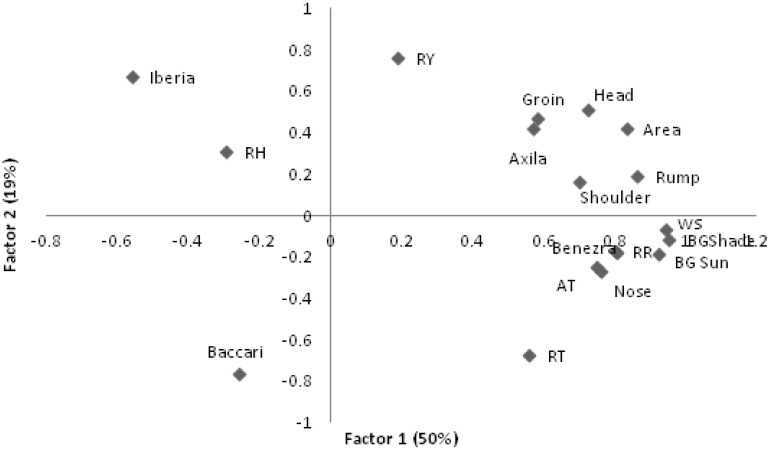
First two eigenvectors for climatic and thermograph measures on lambs. RR–respiratory rate; RT–rectal temperature; RY–Rauschenbach Yerokhin index; AT–air temperature; RH–relative humidity; WS–windspeed; BGTsun–Black Globe temperature in the sun and shade (BGTsd).

BGTshade: black globe temperature under shdade; WS: wind speed; AT: air temperature; *** *p* < 0.0001; ** *p* < 0.001; * *p* < 0.05;

The first two factors (Figure 2) showed that, as air temperature increases, all thermographic measurements increased and Iberia and Baccari indices decreased along with RH. In the second moment, higher AT was associated with increasing RT, RR as well as nose temperatures but this was not accompanied by increasing measurements from the other thermograph measurements. This may indicate a limit to the animal´s ability to lose heat from it surface area while continuing to produce heat internally.

**Figure 3:**
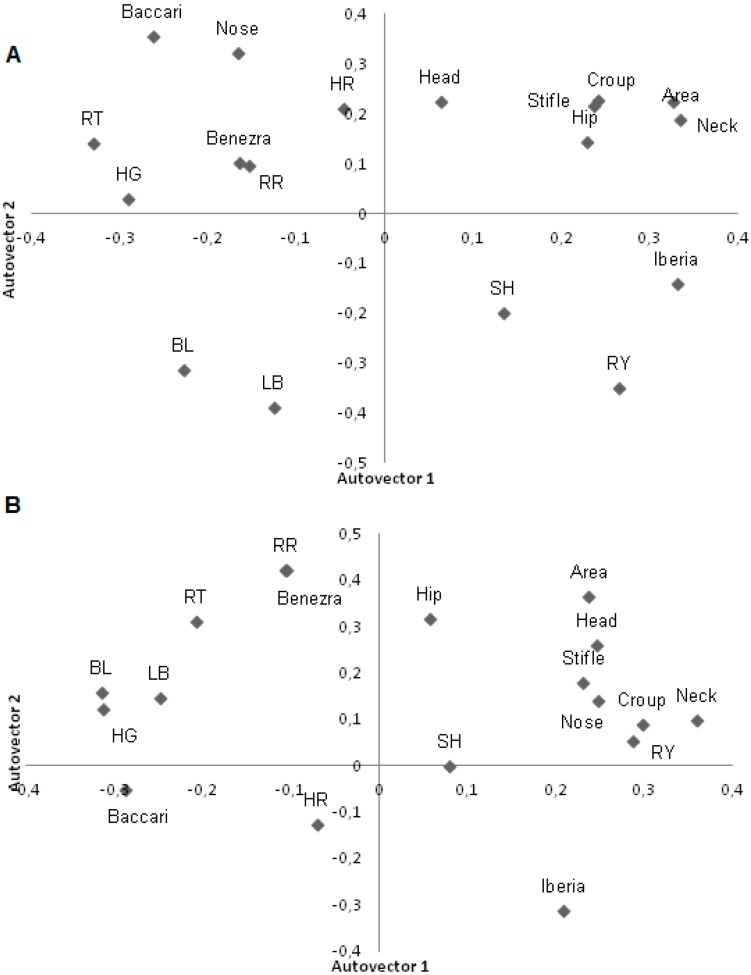
First two principal components for adaptability tests, physiological variables and infrared temperatures in lambs during the morning (**A**) and afternoon (**B**). BL: Body Length (cm); LB: Length of Back (cm); HG: heart girth (cm); SH: Shoulder Height (cm); Flank, Head, Nose, Neck, Stifle and croup: thermography temperatures (°C), HR: heart rate (beats/min); RR: respiratory rate (mov/min); RT: rectal temperature (°C), Iberia: Iberia test; Benezra: Benezra test; RY: Rauschenbach–Yerokhin test; Baccari: Baccari Jr. test.

In the morning, the Iberia and Rauschenbach–Yerokhin tests, which used RT in the formula, were in different components than Benezra which also used RR (Figure 3). In the afternoon, the Baccari index passed to the second factor; that is, increasing RT decreased the Baccari index score indicating lower adaptation of the animal. In the afternoon, RT was close to zero and RR maintained its location, alongside Benezra (Figure 3).

The first two factors for analysis of tolerance traits measured with thermography and carcass traits explained 85% of the variation. Increasing infrared thermography values is accompanied by reduced weight of carcass traits such as weight of the loin, belly, ham and shoulder diameter, and may infer that animals with high surface body temperature have lighter carcasses.

When studying morphology with physiological traits, the first two factors explained 84% of the total variation between the characteristics in the afternoon. The first component separated the size and carcass traits, with little effect on physiological measures. The second component shows that animals with higher values of HR, RT and RR had shorter legs and lighter shoulder cuts.

The discriminant analysis demonstrated the percentage of animals that were correctly classified within each group according to tolerance to heat and physiological parameters such as RT, RR and HR. Generally, in the morning, the animals were not under heat stress (not shown) so comparisons here do not tell us much about the tolerance of these groups. In the afternoon, PRSI scored 100% correct classification using heat tolerance tests and physiological parameters (Table 7). This was the group that showed greater adaptability to tropical climatic conditions in this study. The afternoon increased the impact of infrared temperatures and physiological mechanisms that the animals may have used for maintenance of homeothermy, as the animals were under greater stress in this period.

**Table 7:** Percentage of observations correctly identified (bold numbers) within their genetic group by discriminant analysis for heat tolerance tests and physiological parameters measured on lambs in the afternoon.

	EFSI	PRSI	87PDSI	SI	DOPD	PDSI	WDPD	75PDSI
EFSI	**80**	20	0	0	0	0	0	0
PRSI	0	**100**	0	0	0	0	0	0
87PDSI	25	0	**50**	0	0	25	0	0
SI	20	0	0	**60**	0	0	20	0
DOPD	0	0	16.67	16.67	**66.67**	0	0	0
PDSI	0	0	0	0	16.67	**66.67**	0	16.67
WDPD	0	0	16.67	16.67	16.67	0	**16.67**	33.33
75PDSI	0	33.33	0	0	0	16.67	16.67	**33.33**

PD: Poll Dorset; PR: Primera; EF: East Friesian; SI:Santa Inês; WD: White Dorper; DO: Dorper.

Heart rate was an important discriminating trait, especially for PD crosses. Note that the most frequent type of index used in separating groups of sheep was the Rauschenbach–Yerokhin, developed especially for sheep (Table 8) [13]. The Iberia test, which uses RT, was also important in separating several genetic groups. On the other hand, the Baccari test was important in discriminating the 75PDSI group. This represents the difference between morning and afternoon temperatures for which this group had an intermediate level.

**Table 8:** Physiological parameters and heat tolerance indices responsible for differentiation between eight genetic groups of lambs.

	PRSI	87PDSI	SI	DOPD	PDSI	WDPD	75PDSI
EFSI	Benezra RY	Baccari Iberia	RY HR	Iberia	HR	HR RT	Baccari HR
PRSI		Iberia	RY	Iberia RY	HR RT RY	Iberia HR RY	Baccari Iberia HR
87PDSI			RY Iberia	RY	HR	HR	Baccari RY HR
SI				Iberia RR	HR RT	HR RT	Baccari RY HR RT
DOPD					HR Iberia RR RY	HR	Iberia RR HR
PDSI						HR	HR
WDPD							Baccari RY HR

PD: Poll Dorset; PR: Primera; EF: East Friesian; SI:Santa Inês; WD: White Dorper; DO: Dorper.

When only physiological and thermograph traits were examined (Table 9), most thermograph measures differed between genetic groups showing differing emissions of heat. Rump temperature and RT were the variables that most appeared in genotype separations (Table 9).

**Table 9:** Differentiation between eight genetic groups of lambs using infrared thermography and physiological parameters.

	PRSI	87PDSI	SI	DOPD	PDSI	WDPD	75PDSI
EFSI	RT Rump Head	RT Armpit Rump Neck Head	Head HR RR	RT Stifle Nose	RR Rump Nose	RT Stifle Rump Nose Neck	Rump Head
PRSI		RT Head	RT Rump Neck	RT Rump	RT	RT Stifle Head	RT Stifle
87PDSI			Neck Stifle Flank Head	RT Armpit Rump Stifle Flank	RT RR Rump Armpit Stifle	RR Flank Croup	Armpit
SI				RT Flank	RR Rump Nose	RR RT HR Rump Stifle Flank Neck	Rump Neck Head
DOPD					RT Flank Head	RT Flank	RT HR Rump Head Neck
75PDSI						RT Stifle	Head Nose
WDPD							RR Stifle

PD: Poll Dorset; PR: Primera; EF: East Friesian; SI:Santa Inês; WD: White Dorper; DO: Dorper.

**Figure 4:**
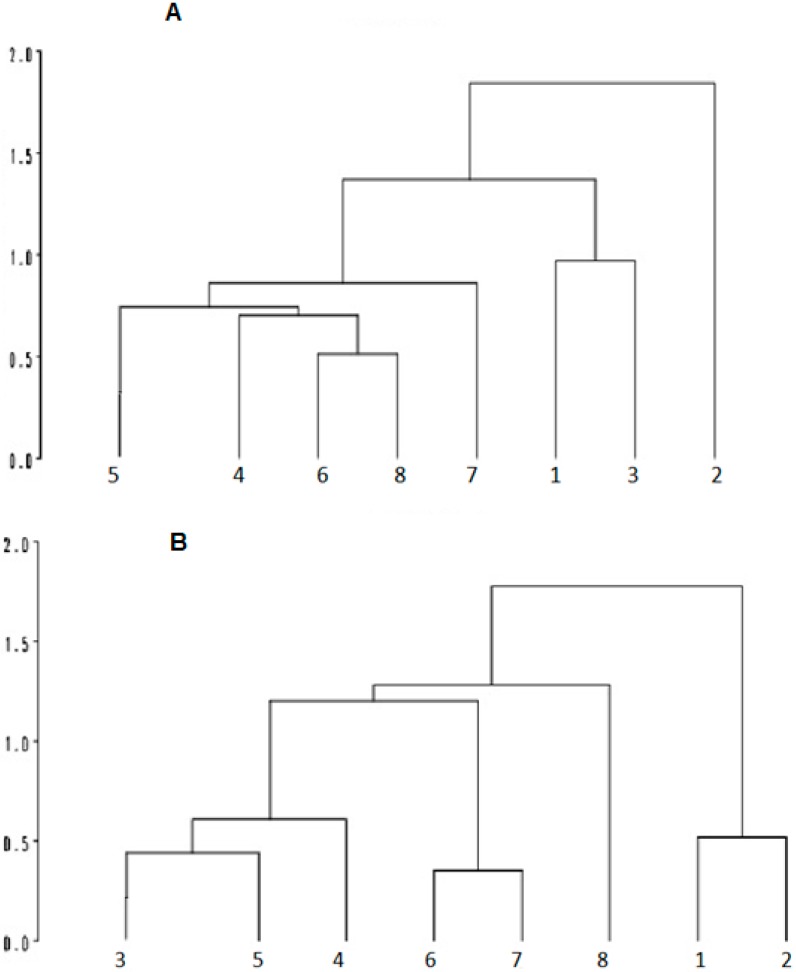
Cluster analysis of variables in the morning (**A**) and afternoon; (**B**) based on thermograph and physiological measures in lambs during the morning (**A**) and afternoon; (B) 1: EFSI; 2: PRSI; 3: 87PDSI; 4: SI; 5: DOPD; 6: PDSI; 7: WDPD; 8: 75PDSI.

The cluster analysis, in the morning, indicated that the SI, PDSI and 75PDSI genetic groups were closely related in terms of response to the environment. All groups contained PD or SI genotypes (Figure 4). The PRSI differentiate from other genetic groups in the morning and afternoon, which is probably related to results obtained in adaptability tests (RY and Ibéria), infrared temperature and rectal temperature in the afternoon, that demonstrate a better adaptation of this group to heat stress.

## 4. Discussion

Animals in stressful environmental conditions use physiological mechanisms to return to homeothermy. If they fail to regain adequate body temperature, they begin to change their behavior, such as reducing food intake, showing a drop in productivity. According to Cunningham [16], changes in physiological parameters are evidence of attempts to move out of the heat stress condition to which the animals have been submitted.

During the afternoon, approximately 52% animals were classified as suffering from high stress, showing that many animals were able to use physiological mechanisms to maintain homeothermy. RR increase was also noted in the afternoon by Brasil *et al.* [17], in goats, and McManus *et al.* [18], in sheep, and RR is commonly used as a measure of heat stress [19]. When air temperature increases, thermoregulatory mechanisms enter in action increasing heat loss through increased sweating and RR [20]. In sheep, the respiratory tract is a primary mechanism for heat dissipation [21] and several studies have demonstrated that this physiological parameter a good indicator of heat stress. McManus *et al.* [18] concluded that the increase in respiratory rate can be considered the primary control mechanism under the environmental conditions imposed, accompanied by other mechanisms, such as increased sweating rate.

Rectal temperature is also a parameter widely used to determine the degree of adaptability of the animals, since an increase of this variable for the species indicates that the animal is storing heat, and so thermal stress may manifest itself [22]. Normal rectal temperatures in sheep vary from 38.5 to 39.9 °C [16]. Some genetic groups showed RT above the reference value indicating difficulty in maintaining homeothermy [21]. Generally, an increase in 1 °C in rectal temperature is enough to reduce performance in most domestic species [23]. The PRSI genetic group had the highest percentage of animals with RT below 39.9 °C (91.18%), indicating that these animals probably suffered less from heat stress.

Rectal temperature in sheep starts to increase when environmental temperature rises above 32 °C [16]. PRSI had the lowest RT (39.43 °C) in the afternoon. These animals may have used respiratory evaporation and peripheral vasodilation to lose enough heat to maintain their body temperature. On the other hand, the EFSI group showed high HR and RR demonstrating low adaptation and, at the same time, had higher thermography temperatures. Probably, EFSI animals had greater increase in blood flow to the body surface to maintain homeothermy, causing an increase in surface temperature [24].

The cluster analysis showing the distance between genetic groups also demonstrated these relationships. The PRSI group differed from others in the morning and the PRSI and EFSI differed from others in the afternoon. In the morning, PRSI seems to be the best-adapted group with low physiological parameters (RT, HR, RR) and infrared temperatures. In the afternoon, the PRSI and EFSI increased their surface temperature to lose heat to maintain their body temperature, as these two groups did not show high RT in the afternoon.

The DOPD had the highest percentage of animals with RT above 39.9 °C (52.78%). Moreover, this group had the highest MCHC corroborating with the inferences that this genetic group suffers with high air temperatures and solar radiation. Long term heat stress can reduce the number of red blood cells [25], directly influencing MCV, MCH and MCHC. Therefore, the DOPD seems be group with the poorest adaptation in this study.

In the Rauschenbach–Yerokhin test, the animals failed to exceed 73.28% efficiency in maintaining homeothermy. Note that air temperature is used for this calculation. The Santa Inês group demonstrated heat intolerance to this (54.48) and the adapted Baccari Jr. (8.32) tests, differing from other genetic groups. The Santa Ines is a Brazilian locally adapted hair breed, which is expected to be well adapted to climatic conditions. However, aiming to improve production traits, the breed recently underwent an introgression process through crossing strategies with specialized meat breeds [8]. This may have promoted losses in adaptation traits [10].

The fact that the pure Santa Ines or crossbred Dorper groups do not distance themselves from the others, especially when a breed that is, supposedly, not heat tolerant is used, makes it clear that the sale of these animals as a well heat-adapted breed is a marketing strategy that farmers should be aware of [2,10,26]. Moreover, further studies should be conducted with these groups since genetic selection for increases in productivity may decrease rusticity and adaptability.

Castanheira *et al.* [27] used multivariate analysis to discriminate genetic groups of sheep, based on physiological and physical characteristics of heat tolerance. Other authors have used multivariate analyses to analyze the distance between genetic groups based on morphological characteristics in sheep and goats, including Herrera *et al.* [28], Dossa *et al.* [29] and Traore *et al.* [30]. As in these, in the present study, size traits were seen to be important in discriminating between genetic groups, as were physiological parameters.

Correlations between thermography, environmental and physiological measures were medium to high and predictive equations show that this method should be efficient in determining respiration rate and rectal temperature in lambs, thereby avoiding unnecessary handling and stress for these animals. The thermography temperatures, mainly flank and rump (high coefficient of determination), yield significant regression with black globe temperature under shade, demonstrating the relation between the thermography and environmental data.

Thermography was shown to be efficient in measuring heat stress in lambs, with medium-high correlations between these measurements and traditional heat tolerance measures such as RR and RT. This has the advantage that it is a non-invasive technique [31,32]. Montanholi *et al.* [33] also found that infrared thermography can be successfully applied for assessing heat and methane production in cattle in an experimental chamber. Their correlations with heat production were in line with those found here for rectal temperatures.

The use of thermal images is a non-invasive technique, which may avoid some of these stress problems involved with estimating heat tolerance in animals without causing interaction though handling. As seen here, it can successfully discriminate between genetic groups and are related to physiological measurements in the animals. These measurements were also seen to be related to red blood series parameters, which are widely used to assess the ability of breeds to adapt, since they are directly involved in mechanisms of heat loss [22].

The main regions of the animal body to measure using thermography seem to be the flank, nose and rump, which is in agreement with results found by Paim *et al.* [13]. Therefore, these points of animal body should be used in further studies using infrared to confirm their usefulness in heat tolerance assessment in lambs.

In recent years, climate change has led to an increase in the number of studies looking at animal welfare, in an attempt to minimize economic losses resulting from the adverse effects of climate on animal production in the tropics [4,13,26]. In this context, new technologies such as thermography could be included to facilitate measurement of stress in animals.

## 5. Conclusions

The assessment of body surface temperature measured by the thermograph is an alternative non-invasive tool for measuring the heat produced and tolerance in lambs. Genetic group is an important factor in heat tolerance and should be taken into consideration when introducing new breeds into production systems. Rectal temperature, heart rate, and thermographic temperature at the rump, flank and nose are important variables for heat tolerance assessment in lambs.
